# Relating spatial perspective taking to the perception of other's affordances: providing a foundation for predicting the future behavior of others

**DOI:** 10.3389/fnhum.2013.00596

**Published:** 2013-09-24

**Authors:** Sarah H. Creem-Regehr, Kyle T. Gagnon, Michael N. Geuss, Jeanine K. Stefanucci

**Affiliations:** Department of Psychology, University of UtahSalt Lake City, UT, USA

**Keywords:** affordances, perspective taking, perception and action, spatial cognition, motor simulation

## Abstract

Understanding what another agent can *see* relates functionally to the understanding of what they can *do*. We propose that spatial perspective taking and perceiving other's affordances, while two separate spatial processes, together share the common social function of predicting the behavior of others. Perceiving the action capabilities of others allows for a common understanding of how agents may act together. The ability to take another's perspective focuses an understanding of action goals so that more precise understanding of intentions may result. This review presents an analysis of these complementary abilities, both in terms of the frames of reference and the proposed sensorimotor mechanisms involved. Together, we argue for the importance of reconsidering the role of basic spatial processes to explain more complex behaviors.

How can different people look at the same object or event and perceive (pretty much) the same thing?… What is even more intriguing is the possibility that I can perceive the meaning afforded by the existing layout of surfaces in the environment for another person as well as for me. What underlies the commonality of perception across diverse individuals? (Mark, 2007, p. 108)

Humans are inherently social beings as evident by the fact that we live in families, work in groups, share meals with one another, relax with friends, and are often entertained by watching the lives of other humans. This is not a new idea, but rather the motivation for establishing the field of social psychology. Furthermore, the “ecological dominance—social competition model” proposed by Alexander (1990), suggests that one of the most influential evolutionary pressures that shaped human intelligence was “…a within-species co-evolutionary arms race in which success depended on effectiveness in social competition” (pp. 4–7). Whether one is trying to gauge an enemy's weakness, or striving to cooperate with a friend, the ability to predict the future behavior of other humans allows actors to adjust their current behavior, providing them with a powerful social advantage (for a review, see Flinn et al., 2005).

Predicting the future behavior of others involves both an understanding of what another person is capable of *doing* and an understanding of their current *goals*. Studies that have explored how a viewer makes judgments of another's action capabilities—other's *affordances*—have revealed that viewers can adequately judge what another is capable of performing when provided information about this others' ability to act (e.g., body dimensions or kinematic information). The ability to take the spatial perspective of another person may provide information about the goals of this other person by revealing their line of sight. While judging other's affordances and spatial perspective taking are often studied under the disciplines of perception and spatial cognition, we propose that these two abilities may also work together to build a foundation for *social* cognition. Our goal is to review the literature from both domains to determine how *spatial perspective taking* and the *perception of other*'*s affordances* work together to predict the behavior of others. In addition, we will review neurological evidence that may provide a biological mechanism common to both processes.

We begin with a review of the behavioral evidence demonstrating that observers have an understanding of what others can do through explicit judgments of affordances for another agent. Second, we review the evidence that spatial perspective taking can reveal the intentions of another agent. Then, we consider how spatial perspective taking and judging affordances for others may be integrated to provide an observer with the information necessary to predict the behavior of others. Next, we consider two distinct but not necessarily exclusive accounts of the underlying mechanisms of social perception and action—motor resonance/simulation (Sebanz et al., 2003; Bosbach et al., 2005; Gallese and Sinigaglia, 2011) and ecological approach/information-based (Marsh et al., 2006; Ramenzoni et al., 2008b). We discuss evidence for the possibility of shared mechanisms with spatial perspective taking and similarities and differences between the way frames of reference are used. Self-judgments are made with respect to the viewer's reference frame (*egocentric*). An important theme is whether judgments about another agent use a transformation of the viewer's reference frame onto the other's egocentric reference frame to update spatial relations (termed *egocentric transformation*), or the use of an *allocentric* frame—the use of relative spatial relations between two points outside of one's egocentric frame. We conclude with a discussion of how both abilities, judging other's affordances and taking the perspective of another, while likely different processes, rely on a social context and support the broader goal of social coordination.

## Perceived self-affordances

Knowing what another person is capable of doing is often considered in the context of the theory of affordances (Gibson, 1979). Gibson's (1979) ecological theory of perception stated that the perception of the environment is directly related to the actions that one is capable of performing in the environment. The term affordance is used to describe the fit between environmental (perceived through the senses) and person features (e.g., size of the body or kinematic capabilities; Michaels and Carello, 1981; Turvey, 1992; Stoffregen, 2003; Plumert et al., 2004). For example, a tree branch lying on the ground can afford sitting, stepping on, or stepping over. A tree branch placed sufficiently higher does not afford sitting or stepping over, but may instead afford walking under. In sum, affordances are opportunities for action present in the environment that are defined by the observer's action capabilities (Turvey, 1992; Stoffregen, 2003).

People are able to judge whether an environment affords a particular action without executing the actual action (termed affordance judgment) and scale environmental features to their abilities (Mark, 1987; Warren and Whang, 1987). For example, Warren and Whang (1987) found that people required apertures to be 1.16 times their shoulder width when judging whether an aperture afforded non-rotated passage. They also found that this affordance was scaled to the eye height of the participant suggesting that the visual information was related to body dimensions and abilities. Other body dimensions are taken into account for other types of actions. For instance, the maximum climbable surface has been found to be about 0.88 times the length of the actor's leg (Warren, 1984; Mark and Vogele, 1988). The critical boundary has been identified for a number of different actions including grasping (Newell et al., 1989) sitting (Mark, 1987), and reaching (Carello et al., 1989).

Affordances can also be learned or recalibrated to fit new capabilities or novel environments (Wagman and Taylor, 2005; Ishak et al., 2008). Ishak et al. (2008) demonstrated that participants were able to recalibrate decisions about whether their hand could fit through an aperture when their hand was made larger. Wagman and Taylor (2005) manipulated the width of participants by having them hold a t-shaped object at their waist. They showed that participants almost instantly recalibrated judgments of passage through an aperture when their body size was widened by holding the pole. They attributed the immediacy of recalibration to the ability of participants to determine the length of the pole by wielding it prior to judgments. Higuchi et al. (2004) investigated the ability of novice wheelchair users to judge their ability to pass through an aperture when in the wheelchair. They found that novice users often judged apertures to be passable when they would not actually fit through in the wheelchair (aperture to wheelchair width ratio of.92). While participants' judgments improved after 8 days of practice with the wheelchair, they did not reach levels observed in baseline performance (without the wheelchair). Under a different paradigm, Mark (1987) and Mark et al. (1990) investigated how an actor comes to know the specific relationship between an environmental extent and their action capability. Mark (1987) altered standing eye height by requiring participants to wear 10 cm blocks underfoot. They then judged their ability to sit on surfaces of different heights. Without practice sitting, participants' judgments of what they could sit on returned to the critical boundary when not wearing blocks over the course of 30 trials. Mark et al. (1990) then systematically manipulated information available to the participant when wearing the blocks. They found that participants were able to recalibrate their judgments of sitability to their new height when they were able to locomote, move their heads or eyes, or lean to the side. Restricting visual information by providing only monocular viewing through a peephole or restricting movement by requiring participants to rest their heads against a wall significantly reduced participants' ability to recalibrate information and judge sitability with blocks underfoot.

This body of work is important because it shows that people are fairly accurate in judging what they are capable of performing in an environment. This work also demonstrates that people are able to quickly calibrate their affordance judgments to changes in their ability to act. The rate of recalibration is often determined by the degree to which observers experience or gain information about the change to their capabilities. Others have theorized that flexibility in affordance judgments and the performance of actions is necessary to deal with changes in the demands of the situation, changes to the criteria for success (the goal), and changes with the availability of visual information (Fajen et al., 2009). Importantly, this work demonstrates that all of the information necessary to judge and carry out an action is available to the person in the ambient stimulus arrays in which the person is immersed.

## Perceiving others' affordances

As introduced above, affordances for the self are typically grounded in an egocentric frame of reference and scaled in terms of one's body dimensions with respect to the current viewpoint. However, when judgments of other people's affordances are made, it is possible that observers switch to an allocentric frame of reference. We define allocentric judgments as those that are relative judgments made between two points, outside of the self. As such, the environment is scaled to the other's body rather to one's own (Stoffregen et al., 1999). Rochat (1995) examined reaching affordances of children and adults, asking whether young children distinguish reachability for themselves and others. The findings revealed that both children and adults scaled their judgments of reaching to their own physical characteristics in the self-judgments and to the other's physical characteristics for judgments of the other. In addition, all subjects showed the ability to take into account the other's change in reaching height when viewing the other on “tip-toes.” These findings suggest an early ability to switch from an egocentric to an allocentric frame of reference in this task. More recent studies with adults have focused on judging others' affordances when the action involves either a single other person or the potential actions of dyads (the observer and another person).

### Affordances for one other

For single-person affordances, multiple studies have shown that observers accurately scale environmental features to the action capabilities of the actor being observed (Stoffregen et al., 1999; Ramenzoni et al., 2008b,c, 2010). Stoffregen et al. (1999) examined observers' abilities to perceive the maximum height at which another actor could sit. In this extensive study, the observer judged their own and another actor's affordances for sitting, while varying the height of the other actor as well as the viewer's experience with observing kinematic displays of the other actor perform non-sitting actions. They found that affordances of others were scaled with respect to the actor's leg length. In addition, Ramenzoni et al. (2008b) tested judgments of maximum reaching height of the self and another with the goal of testing whether eye-height information would be used. Observers judged how high they or a different sized actor could reach an object while the observer stood on the floor, or one of two different sized steps. The other actor always remained standing on the floor. Judgments, when scaled to the observer's reaching height for the self and to the actor's reaching height for the other, were near 1.0, indicating that estimates were very accurate, both for self and other. These results support the notion that affordances are scaled to the intrinsic units of the observer (in self-judgments) or actor (in other judgments). Mark (2007) summarizes a series of studies following up on these findings, replicating the effect for sitting, climbing, and stepping affordances. These studies argue for the claim that an allocentric frame of reference is adopted when judging affordances for others and that observers can do this in the context of judging their own affordances as well-switching easily from an egocentric to an allocentric framework.

Some actions, like jumping-and-reaching, require the observer to have information about the actor's kinematic abilities and not just information about the size of the actor (Weast et al., 2011). Stoffregen et al. (1999) found that when observers were provided with the appropriate information about the underlying dynamic actor properties, they could accurately judge the other's ability. In addition, Ramenzoni et al. (2010) asked whether a learning paradigm would influence maximum jump to reach estimates for another actor over multiple repeated trials in a similar manner as self-judgments. They found an increase in accuracy across trials for self-estimates, but not for actor estimates. The lack of changes over time in the other's judgments suggests that judgments of others are not dependent on judgments of self. However, their second study tested the influence of watching the actor perform a task related in dynamics (lifting) or unrelated to the dynamics of jumping and reaching (torso-twist) on judgments of reach-by-jump for both the self and the other. They found that watching an actor perform a related task improved the accuracy of the estimates of the actor's capabilities but watching the unrelated task did not help. The second experiment showed that experience with another's kinematic abilities facilitates related affordance judgments, suggesting the importance of calibrating the observer to specific action-relevant information about the actor's capabilities. Weast et al. (2011) investigated how expertise influenced the perception of affordances for others. They found that basketball players were better than novices at judging the jump-and-reach height of another actor but that basketball players were no better than novices at judging a non-sports-relevant action (sitting height). In their second study, they demonstrated that with exposure to kinematic information, basketball players', but not novices, judgment of maximum jump-and-reach improved. This finding suggested that basketball players had enhanced sensitivity to kinematic information. These findings emphasize the claim that the relationship between the other's physical body parameters (e.g., size and capabilities) and the environment, as well as degree to which someone has experience with a specific relationship, is critical in informing decisions about others' ability to act in the environment.

Another series of studies examined the ability and accuracy of adults to judge reachability of children (Cordovil and Barreiros, 2010, 2011) generally supporting the claim that observers scale affordances to the other's body, but also showing less consistent overestimation in judgments of children's reaching compared to adult self-judgments. As in Ramenzoni et al. (2010); Cordovil et al. (2013) asked whether accuracy in judging another's affordance may be a function of experience or practice. Cordovil et al. (2013) tested adults' judgments of the maximum standing reachability, reach and jump reachability, and step-length of a 5 year old boy, before and after observing the boy perform the action. They found that viewing the boy's actual affordance improved the more complex affordances (jump-to-reach and step-length) but had little effect on the basic reaching while standing judgment. The observation/practice manipulation suggests that when given more information about the relationship between the other actor and the environment, observers can calibrate the information to adjust their response.

A somewhat different take-home message comes from Ramenzoni et al. (2008a) in a study of perceived maximum reach by jumping. The observer's capability to jump was manipulated by wearing ankle weights. Judgments were made both for the self and for another actor who did not wear ankle weights. Interestingly, estimations of jumping-reach height were lowered not only for the self, but also for the other actor, specifically after the observer walked while wearing the weights. The effect of ankle weights to reduce the critical boundary of reach by jumping is consistent with the body of work showing that effort or behavioral potential influences spatial judgments (Proffitt, 2006). However, what is unique about these findings is that the manipulation affected judgments of what someone else could do. These results support a social context underlying perceived affordances and suggest that judging others action capabilities may rely somewhat on how the observer herself can act. Thus, the task becomes one at least partially based in the observer's egocentric frame of reference. Notably, in this study, observers may not have had sufficient information about the actor's jumping ability to rely solely on the relationship between the other and the environment to make their judgment. The influence of the ankle weights on judgments of the actor's capabilities may be erased if sufficient information about the actor's kinematic capabilities is provided.

### Affordances for dyads

Another way in which researchers have assessed the ability to judge others' affordances has been to examine dyads or joint actions. This work looks at how observers are able to make decisions about actions when these actions are to be performed in correspondence with another person. This is especially interesting because, unlike the single-person judgments, observer and actor actions necessarily have a direct influence on one another. Further, different action capabilities may result as two observers coordinate their actions (Isenhower et al., 2010). Chang et al. (2009) took this approach in an environment-person-person system, testing whether adults would accurately estimate their ability to pass through an aperture while walking through with a child. The adult and child were attached with a Velcro strip at the child's elbow and the adult's wrist. The results showed that adults were able to accurately perceive affordances for passage with the child. Consistent with the self and single-actor studies, the results revealed that judgments were scaled additively to the intrinsic units of the adult shoulder width + child shoulder width.

Similarly, Davis et al. (2010) assessed how two adults performed the joint action of walking through an aperture. First, they established that a “shared” model, rather than an additive model, better predicted the critical boundary for the dyad's actual passage. This showed that the critical aperture width was less than the sum of the critical aperture widths for each actor separately suggesting that coordinated actions are scaled to the combined action capabilities of the two actors. Further, they examined the influence of action-observation experience on perceived affordances for passage of the self and the other actor. Participants either viewed the other actor walk, walked alongside the actor, or viewed the actor standing only. As in previous work, the ratio of critical width to actual shoulder width (scaled to the participant for self-judgments and the actor for other judgments) were nearly identical, suggesting the ability to use the other's intrinsic scale to make estimates. However, the dyad estimates were significantly underestimated with respect to the actual joint affordance. Furthermore, unlike some of the previous work, there was no effect of the increased action-observation conditions. The reduced accuracy in response is similar to the person-plus-tool studies mentioned above (Higuchi et al., 2004; Wagman and Taylor, 2005), and is likely a result of insufficient information or lack of experience walking as a dyad.

The body of literature on perceiving affordances for one other and for dyads suggests that observers are capable of judging what another person can perform. These judgments are likely completed by using an allocentric frame of reference, and they reveal what actions another person is or is not capable of performing in the current environment. In addition, an observer's judgments about another person are scaled to the action capabilities of the other person or the other person + self system. When making judgments about actions that require more than a relative size comparison, observer's judgments about another's affordances improve when they see the actor perform similar dynamic movements. There is also evidence that when an observer is not provided with kinematic information about the actor that the observer may use their own ability as a baseline to judge what another could perform. Notably, much of the existing literature involves judgments of others in tasks such as walking through apertures that does not involve critical time constraints. It may be that in more interactive dyadic tasks, such as lifting a box together, different information relevant to action coordination is used (see later section on synergistic accounts). In all, the evidence points to the use of an allocentric frame of reference generally used for perceiving other's affordances, with the influence of an egocentric frame of reference when there is insufficient information available about the other's capabilities.

## Other's affordances: summary and conclusion

There is clear evidence for the human ability to judge what others can do, as well as to use what others can do to influence their own action judgments. Together, this work reinforces the idea that others' affordances are used as an important component in the broader problem of predicting the future behavior of others. However, if humans only had at their disposal the ability to judge action capabilities for another, they would have to consider all of the affordances that a given environment offers to this other person. This would be a rather cumbersome way to predict the behavior of others, unless there was a meaningful way to focus on only a few affordances. The theory of affordances (reviewed above) may provide some insight to this problem. When perceiving affordances for oneself, observers orient their senses to the properties of the environment that are necessary for perceiving a particular affordance. For example, if someone intends to grasp an object sitting atop a tall shelf, they will likely look in the direction of the object. If they can reach the object they will then do so, otherwise they will likely look around for a heightened surface that affords standing/climbing and will use this surface to reach the object. Therefore, assuming that other people also orient their senses to pick up information relevant to a potential action, an observer can simply identify where this other person is looking and consider the actions that this spatial location may afford for the other person. Much of the research that has examined our ability to detect where another person is looking, what they can/cannot see, and their spatial relationship to other objects in the environment is called spatial perspective-taking and will be reviewed next.

## Spatial perspective taking

Research on spatial perspective taking has a long history across both developmental and cognitive psychology ranging from Piaget's classic three mountain task (Piaget and Inhelder, 1967) to a comparison of physical and imagined body rotations (Rieser, 1989). The role that spatial perspective taking plays in spatial memory and navigation has also been examined (Loomis et al., 1999; Shelton and McNamara, 2004). Perspective-taking research is also interested in how observers determine what another person can or cannot see, and is often called joint (shared) visual attention (Frischen et al., 2007). In general, spatial perspective taking encompasses a class of phenomena that involve accessing spatial information relative to a viewpoint different from one's own egocentric viewpoint. Importantly, we will examine whether these abilities may allow an observer to suppose the intentions of another person.

Spatial perspective taking can be differentiated into Level-1 perspective taking (PT-1) and Level-2 perspective taking (PT-2) based on developmental stages and proposed underlying processes (Salatas and Flavell, 1976; Kessler and Rutherford, 2010). PT-1 is often defined as a visibility task in which an observer determines what another person can or cannot see. One of the first studies examining this type of task with adults was aimed at establishing shared common ground in a virtual environment. Kelly et al. (2004) asked observers in the real world or in a virtual environment to judge whether another agent could see a given target in the environment. The scene was purposefully chosen (or created in VR) so that there was an occluding building, and the viewer was given instructions to judge which parts of the scene were visible from the other's viewpoint and which were occluded by the building. They indicated this on a photograph of the scene (in the real world) or by pointing to the location in the virtual world. Viewers were generally good at this task across both environments, but overestimated what the agent could see as the distance between the viewer and the agent increased from 5 to 10 to 15 m. This work suggests that PT-1 may utilize an allocentric frame of reference in which observers visually match various distances and angles to infer the line of sight of another.

In contrast, PT-2 typically requires an observer to identify where in space a target object is located relative to a viewpoint that is different from the observer's current viewpoint. For example, in early work on imagined and real transformations, Rieser (1989) asked participants to learn the location of an array of objects while standing in the middle of the array. While blindfolded, they were asked to point in the direction of a named target from a new imagined viewpoint. Then they were asked to imagine facing in a new direction (rotation task) or to imagine moving to a new target location while continuing to face in the same cardinal direction (translation task). This and other work (e.g., Presson and Montello, 1994; May, 2007) showed a robust angular disparity effect in the imagined rotation task, such that reaction time increased with the increasing disparity between one's actual facing and imagined facing direction. This was significantly different from the virtually flat response time function found in real rotations, suggesting a cost to perform the mental transformation to judge what the spatial layout looked like outside of one's physical viewing perspective. From this work, Rieser (1989) and Presson and Montello (1994) suggested that the angular disparity effect found in PT-2 tasks is due to the increased processing involved in updating self-to-object relationships.

May (2004, 2007) suggested that the angular disparity effect may be due to a conflict of sensorimotor codes. Specifically, a conflict in sensorimotor codes occurs between codes that help identify the location of a target object from the to-be-imagined viewpoint, and the codes that help the observer actually make a pointing response. This was initial evidence that PT-2 involves a shift from one egocentric frame of reference to another egocentric frame of reference. Kessler and Thomson (2010) provided additional support for the use of egocentric reference frames during PT-2 by showing that the observer may actually imagine rotating her body axes to align with the to-be-imagined perspective. They asked participants to indicate whether an object was located to the left or the right of an avatar situated at 0, 40, 80, 120, or 160° around a circular table with respect to the participant's viewpoint. Importantly, the authors situated the participants at the computer such that their bodies were either facing straight ahead toward the monitor, or at a 40° angle from the monitor. They found an overall effect of body posture that increased monotonically with angular disparity. In other words, observers switch from their current egocentric viewpoint to the egocentric viewpoint of another person in space in order to mentally transform their body axes through the space. May and Wendt (2012) have more recently pointed out that some egocentric mental transformation tasks also face stimulus-response compatibility effects, where spatial conflict may contribute to the apparent mental transformation effects.

Overall, the difference between visibility tasks (PT-1) and determining spatial relationships from a new perspective (PT-2) may be the object relations that are used. Inter-object relations may be used to determine whether something is visible from another's perspective. However, when updating to a new left/right position respective to that perspective, rotation of the viewer's frame of reference is needed. In support of this claim, several have found that left/right decisions involve increasing response time with increasing angular disparity, whereas visibility/front back decision show relatively flat response time functions as a function of angular disparity but increasing response time as a function of distance between the agent and the target (Michelon and Zacks, 2006; Kessler and Rutherford, 2010). In summary, PT-1 appears to rely on an allocentric frame of reference, determining the location of an object with respect to another's viewpoint whereas often PT-2 relies on the *transformation* of the egocentric reference frame onto the other's viewpoint, in order to update object spatial relations with respect to the new viewpoint.

### Others and spatial perspective taking

Both PT-1 and PT-2 can contribute to a viewer's ability to predict the behavior of others. Several examples come from the study of spatial language in which different frames of reference may be used to produce spatial descriptions to a partner depending on the social context. Generally, these studies show that attributional cues about the partner influence how people interpret and produce spatial descriptions. When speakers perceive that partners have less knowledge or relevance to the task—due to a number of factors such as lower spatial abilities, less familiarity, less agency, or less information about the viewpoint—then speakers are more likely to take a partner-centered frame of reference (Schober, 2009; Duran et al., 2011; Galati et al., 2013). In other words, when the observer realizes there is less of a shared perspective, they will adjust their language to meet the needs of the partner. When the partner's goals, realism/presence, or shared mutual understanding increase, then speakers are more likely to use their own egocentric perspective.

Further, in a simple, but elegant manipulation of the visual presence and goals of an agent, Tversky and Hard (2009) showed that the presence of another person in a scene changed the way people described the left/right relationship between two objects. Observers viewed a photograph of two objects on a table, with or without a person seated across the table either looking at or reaching for one of the objects. The frequency of reporting the relationship of the two objects from the other's perspective increased with the presence of the person, and increased further when the question referred to action. These results suggest that even outside of an explicit communication task, viewers will spontaneously take the perspective of another person. Spontaneous perspective taking was also seen in Samson et al. (2010), who required a viewer to judge (in a picture) how many discs on a wall could be seen from their own perspective or from an avatar's perspective (a PT-1 visibility task). The number of discs that the avatar could see was either consistent or inconsistent with the number of discs from the viewer's egocentric perspective. Viewers were slower to make their egocentric judgments when there was a conflict with the avatar's perspective, even though the avatar perspective had no direct relevance to their task.

Consistent with these results, implicit perspective taking has also been shown with an action-based mimicry task. For example, participants viewed a virtual tight-rope walking avatar while they were simultaneously asked to imagine also being on a tight-rope (Thirioux et al., 2009). The participants were told to lean the way the avatar was leaning, not specifying whether to lean as if the avatar was a mirror reflection, or to lean as if they were in the shoes of the avatar. The study found that the participants adopted the viewpoint of the avatar instead of mirroring the avatar nearly 70 percent of the time.

Many of these studies tend to naturally confound body orientation or depicted action with eye gaze. Mazzarella et al. (2012) decoupled action and eye gaze in stimuli depicting another agent to assess when perspective taking would occur. In contrast to Tversky and Hard (2009), they first used an explicit perspective taking task in which participants were instructed to report target location from either an egocentric perspective or the agent's perspective. Participants viewed scenes with an agent positioned across the table with an object. The scenes varied as to whether the agent looked at or grasped the object. Given the explicit task of taking an egocentric or allocentric frame of reference, it is not surprising that viewers made few allocentric errors in the egocentric condition. However, the results also showed that in the explicit allocentric condition, viewers were better in their allocentric judgments when the actor was depicted as grasping the object, with no significant influence of eye gaze. A third experiment distinguished between the effects of grasping and gaze on perspective taking and attentional orienting. When the task was to detect an object after being presented with the agent-in-action/agent-gaze images, participants were faster with the gaze image than the action image. These results suggest that gaze and body/action information may provide different information about others' intentions. Arm/body cues may be more useful in communicating current goals and eye gaze may indicate what the actor will do in the future.

## Spatial perspective taking with others: summary and open questions

Overall, the work reviewed on spatial perspective taking with others describes two types of tasks, Level-1 and Level-2, which are both elicited in the context of another agent. First, this work suggests that observers may identify the intentions of another by considering where they are looking (PT-1). Second, this work suggests that the body of the other may indicate current goals of the actor while the eye gaze of the actor may denote future goals. Both could be used to understand the intentions of others. Finally, the work reviewed suggests that PT-1 uses an allocentric frame of reference while PT-2 involves shifting from one egocentric reference frame to another's egocentric reference frame.

Much of the spatial perspective taking research has been designed to understand spatial memory, language, navigation, and overall spatial cognition. However, very little of this work has considered the broader social function of spatial perspective taking—predicting other's behavior in the service of coordinating actions. If spatial perspective taking operates in conjunction with perceiving affordances for others, it may have evolved to help us infer an intention or goal for another person. When used alongside the ability to judge this other person's action capabilities, both may allow humans to make fairly accurate predictions about what another person is likely to do next. In turn, observers are able to adjust their own actions to coincide, cooperate, or compete with another person's current and future behaviors.

## Spatial perspective taking and perceived action capabilities mutually inform behavior prediction

### How level-1 perspective taking and judging affordances for others may work together

Gibson (1979) argued that all of the information necessary to judge affordances is available to any point of observation (see also Stoffregen et al., 1999; Mark, 2007). Likewise, information specifying one's line of sight is also available in the optic array. Both PT-1 and perceiving affordances for others utilize an allocentric frame of reference because both processes can be carried out using object-to-object relationships, likely with a visual matching strategy. Although it is unknown how humans (or other species) determine where another is looking, it is plausible that visual information regarding the direction of one's gaze is combined with perceptual information identifying the distance and depth of objects in the environment (see Kelly et al., 2004 for a similar view). Together, it may be possible for an observer to see another person and simultaneously know (1) where they are looking and (2) what actions they are capable of performing given the properties of the environment. This would suggest that the line of sight operates to orient the observer's attention to the properties of the environment that must be considered alongside the bodily capabilities of the other person. Such a process is consistent with Kugler and Turvey's (1987) definition of an intention being an attribution that an observer projects on to another person to simplify what behaviors might be expected from this person. They use an example in physics, in which temperature and pressure are concepts used to understand collective properties of molecules. The temperature of a substance is attributed to the molecules by the observer in an attempt to describe higher level processes when describing the individual movement of each molecule is cumbersome. Much the same, attributing intentions to an actor, is a method by which an observer attempts to reduce the many possible actions available to an actor to a subset few and in so doing describes the demands of the environment that are placed on the actor. Future research should consider testing the possibility that Level-1 perspective taking occurs when attempting to predict the behavior of others.

### How level-2 perspective taking and judging affordances for others may work together

Level-2 perspective taking is distinguishable from Level-1 based on the extent to which observer-centered spatial transformations are needed (as discussed above). PT-2 reveals to an observer the spatial relationship between a person and objects in the environment. For example, you can sit across the table from a friend, and while your friend's cup may be on your right-hand side, you are able to identify that the cup is on your friend's left-hand side. There are many different models that attempt to account for this ability to discriminate one's own perspective from another. Overwhelmingly the evidence suggests that the observer must imagine a rotation of their body axes or frame of reference, possibly involving the motor, proprioceptive, or vestibular system to accomplish this task (Grabherr et al., 2007; Kessler and Thomson, 2010). PT-2 requires that the observer transform their own egocentric frame of reference to the egocentric frame of reference of another person. This is different from how reference frames are utilized when perceiving affordances for others, as judging another's affordances likely involves a shift from the observer's egocentric frame of reference to an allocentric (other-to-object) frame of reference.

Regardless of the use of different frames of reference, the intentions of another actor may still be inferred through PT-2 when an asymmetry exists between the other's left and right side. For example, if another person is holding a rod in their right hand, their ability to reach to objects differs for their right and left sides (Linkenauger et al., 2009). Thus, one could infer that the actor is more likely to reach with her right hand, an understanding that may be critical for a task involving joint action. However, when a distinction between what is on the left or right of an actor is not needed, PT-2 processes are not likely relied on for judging affordances of others. Instead, the observer can visually match the length of the actor's arm (or arm plus rod) to the distance between the actor and some object, thereby inferring what the actor can do by using an allocentric reference frame from the observer's viewpoint. However, PT-2 perspective taking could be integral for successful communication in which two or more people need to create a common conception of the space (Duran et al., 2011). In addition, PT-2 perspective taking appears to be closely related to path integration during navigation, and developing a geocentric view (bird's eye view) of the space (Loomis et al., 1999). In conclusion, it may be the case that PT-2 perspective taking is not used when determining the intentions of other people unless future coordination is required.

## Self and other affordances mutually inform behavior prediction

There are instances in which information about the observer may be used to understand the capabilities of another, and conversely, instances where the capabilities of another influence actions or judgments about the self. For example, in joint action, previous research suggests that observers consider not only their own action capabilities, but also the action capabilities of another person (Sebanz et al., 2006). Even when joint action is not an explicit goal, recent evidence suggests that judging affordances for oneself can be influenced by the action capabilities of another person (Gagnon et al., in preparation). In our own recent work (Gagnon et al., in preparation) we examined both the influence of one's own body size on affordance judgments for another, and the influence of another's size on self-judgments. In a paradigm using judgments of passage through apertures, we found that the judgments for another are scaled to the other's body size, but that there is an additional mutual influence of the self on other judgments and the other on self-judgments.

In addition, Costantini et al. (2011) tested the influence of the affordances of another agent on the spatial alignment effect paradigm (Bub and Masson, 2010)—an effect showing that action-relevant but task-irrelevant objects will facilitate actions when the object is congruent with the action. Previous work showed that in a desktop virtual environment, the presentation of a mug facilitated a grasp response, but only when it reachable by the actor as depicted in the virtual scene (Costantini et al., 2010). Costantini et al. (2011) extended this paradigm and found that the viewer's motor facilitation also occurred when the object was outside of the viewer's reachable space but *within an agent's* reachable space. They suggest that the space in which the actor can perform an action might be “mapped on” to the observer's bodily spatial representation, influencing the observer's own potential to act. This could inform an observer about how another agent perceives a space and capability for action, as well as providing information for joint action (Costantini et al., 2011).

Related spontaneous use of another's potential for action has been demonstrated in a distance judgment task that varied the extent to which another agent could reach a target (Bloesch et al., 2012). Bloesch et al. proposed that if using a tool makes a distance appear closer (see also Witt et al., 2005), then it may be that watching another agent use a tool also influences perceived distance. These predictions held true; observers who watched another actor reach successfully to a target with a reach-extending tool judged the distance to be closer than those who watched an unsuccessful arm-based reach.

As social beings, the mere presence of another person may prompt humans to share (implicitly or explicitly) spatial and proprioceptive information with each other. Oullier et al. (2008) found that when two people see each other performing the same action, they spontaneously synchronize their actions, suggesting a means of information exchange that could coordinate actions. Whether these examples are an instance of a transformation of one's egocentric frame of reference is unknown. Regardless, this work suggests that spatial and proprioceptive information is not necessarily confined to the physical boundaries of a person, but can be shared amongst two or more people.

## Possible overlapping mechanisms supporting other's affordances and spatial perspective taking

Given the relationship between judging others' affordances and spatial perspective taking is somewhat unclear from the behavioral work, it may be useful to consider whether the process of judging other's affordances and spatial perspective taking share overlapping processes relying on motor simulation. First, we will review the proposed mechanisms involved in perspective taking, and then relate this to the potential mechanisms involved in perceiving affordances for others.

### Mechanisms for spatial perspective taking

One explanation for the angular disparity effects present in spatial updating after imagined rotations is sensorimotor interference. Despite evidence for the need for mental transformation of the egocentric reference frame (Rieser, 1989; Presson and Montello, 1994; Easton and Sholl, 1995; Wraga et al., 2000), costs in perspective taking have been attributed to a response-based conflict between one's real and imagined perspective. This is especially apparent in pointing tasks where the correct response is incompatible with the viewer's current physical proprioceptive information for facing orientation (Wraga, 2003; Avraamides et al., 2007) and has been shown to be reduced by disorienting participants before the response (May, 1996). Taken together, this work suggests that sensorimotor processes may underlie spatial perspective taking given the disparity in imagined and real locations influences task performance.

Recent work suggests the influence of the vestibular system in imagined perspective taking as well (Mast et al., 2007). For example, van Elk and Blanke (2013) asked participants to perform imagined viewer rotation while being passively rotated clockwise or counterclockwise. By passively rotating the participants the authors were able to separate some of the proprioceptive cues used in active rotation from the vestibular signals. When the participants were being passively rotated in the same direction that they imagined rotating their viewpoint, reaction times were faster than when the passive rotation was incongruent to the imagined rotation direction. Grabherr et al. (2011) compared patients with unilateral and bilateral vestibular loss on egocentric and object mental transformation tasks. They found that those with bilateral loss showed significantly poorer performance in the egocentric transformation task than unilateral loss patients. In healthy participants, galvanic vestibular stimulation (GVS, direct electrical stimulation of vestibular end organs) has been shown to lead to poorer performance on imagined viewer rotation (Grabherr et al., 2007; Lenggenhager et al., 2007; Dilda et al., 2012).

There are other accounts that may better explain certain types of perspective taking tasks, such as the visibility tasks (PT-1) described above. For example, there is evidence that for visibility tasks, judgments about what another can do may be solved based on visual-spatial processing that do not require a shift to an imagined viewpoint (Kelly et al., 2004; Michelon and Zacks, 2006; Kessler and Rutherford, 2010). Predicting whether an object is visible from another agent's viewpoint is likely performed without a transformation of one's egocentric frame of reference. Rather, the answer can be computed based on an object-to-object based strategy, where a mental line is constructed from the agent to the target. While a viewer-based transformation could be used to solve the task, the lack of an angular disparity effect suggests that the line-of-sight computation is used. There is little evidence in support of any body-based simulation underlying this type of judgment. An open question for the current paper is how mechanisms for spatial perspective taking may or may not be related to affordances and how they may work together to coordinate action.

Several of the mechanisms proposed for spatial perspective taking involve sensorimotor processing. Likewise, one dominant account for the understanding of other's actions—particularly the observation of other's overt actions—is also framed in the motor system. If perceiving other's affordances and spatial perspective taking rely on similar mechanisms, then this suggests that they may be functionally related with respect to social coordination. While on one hand motor simulation may underlie both processes, we must concede that it is possible that it does not account for either process. As described above, there is relatively strong support for the use of perceptual information available to the other, not the self, in judging other's affordances. Further, there is evidence that non-motor, visual-spatial processing may be used for at least some Level-1 (Kessler and Rutherford, 2010) and Level-2 (Amorim et al., 2006; Creem-Regehr et al., 2007) perspective taking tasks. We consider the evidence for both motor simulation and non-simulation/visual-information based accounts of perceiving other's affordances below.

### Mechanisms for perceiving affordances for others

Gibson's (1979) concept of affordances and much of the work following this theoretical viewpoint was concerned with characterizing perception at the level of the observer-environment system. As with any psychological process, one may ask how the process is supported by our biology. While the theory of affordances did not attempt to address questions about the underlying neurocognitive mechanisms involved, there is a related notion of object-based affordances, alluded to in the work of Costantini et al. (2011) above, which elicits motor system activation and could help to explain the mechanisms underlying the prediction and use of other's affordances. Numerous studies with objects have shown that affordances may be automatically activated and lead to subsequent effects on the motor system. For example, a classic behavioral study by Tucker and Ellis (1998) showed a response compatibility effect. When presented with images of objects with handles, responses to an irrelevant stimulus feature were facilitated when the handle orientation was congruent with the hand used to make the response. Neuroimaging has supported this claim, showing that activation of related premotor and parietal cortex results from simply viewing objects such as tools that have affordances (Chao and Martin, 2000; Creem-Regehr and Lee, 2005). It is important to note, however, that goal context has been shown to be important in modulating activity across both cognitive and neural approaches. Buxbaum and Kalenine (2010) provide compelling examples of how motor resonance may only occur in the context of goal-directed, functional representations of objects, rather than simply the structure of the object itself (see also, Creem-Regehr et al., 2007). Can the neurocognitive notion of object affordances (mostly focused on grasping) be extended to environmental affordances such as those for passing through and sitting? We discuss this possibility in terms of motor resonance theory below.

### Motor resonance

The “mirror neuron” system is a specific brain mechanism proposed to underlie motor simulation during action observation. Mirror neurons were identified initially in the ventral premotor and parietal cortices of the macaque monkey. They activate both when the monkey performs an action as well as when the monkey observes another human or monkey perform the same action (Gallese et al., 1996; Rizzolatti and Craighero, 2004). A body of work has proposed an analogous system in humans, including the premotor cortex, inferior parietal cortex, and superior temporal sulcus, with specificity to the level of somatotopic representation of specific body parts (Buccino et al., 2001) and tuning to the actual motor capabilities and experiences of the actor (Calvo-Merino et al., 2005, 2006). Subsequent research has defined some mirror neurons as goal-related rather than effector-specific (Fogassi et al., 2005; Rochat et al., 2010). For example, Fogassi et al. (2005) found mirror neurons in the monkey inferior parietal lobule that responded to observation of the same grasping action differentially *as a function of the goal of the action*. Neurons were selective for the goals of grasping-to-eat vs. grasping-to-place. Similarly, in humans, Iacoboni et al. (2005) varied whether an observed grasping movement was performed in the context of goals of drinking or cleaning up. Premotor cortex activity was modulated by the context and intention of the grasp depicted. The importance of understanding a hierarchy of goals has been emphasized by several researchers (Grafton and Hamilton, 2007; Thill et al., 2013). Also, when performing a joint action, there is neural activity associated with coordinated (phi 2) and independent (phi 1) behavior. Topographically, this activity maps well to the mirror neuron system, and phi 1 (independent behavior) may indicate inhibition of the mirror neuron system (Tognoli et al., 2007). Many have proposed that we understand the actions of others by means of a motor or embodied simulation system, although these claims have also stirred much debate. How then, might this mirror system support the judgments of what others can do and see?

The term motor resonance refers to the matching of one's own action to another's (Uithol et al., 2011). As Uithol et al. (2011) described, the term “resonance” comes from the physical phenomenon that two systems oscillate and at the same frequency and phase as one another. However, in the neurocognitive context of mirror neuron systems, resonance is used more broadly to describe a mechanism of emulation, in which viewing an action performed by another leads to activation of neurons in the viewer that represent that action. Viewers understand actions by matching or simulating the action. Furthermore, the analysis by Uithol et al. (2011) differentiates between intrapersonal resonance and interpersonal resonance—a distinction that may be important for the extension to judging other's affordances. Intrapersonal resonance occurs within an individual: a perceptual representation of observed action is activated and at the same time coupled with a motor representation (Rizzolatti et al., 2001). This notion is supported by the common coding theory (Hommel et al., 2001) in which perception and action share common underlying representations. In interpersonal resonance, there is a functional equivalence between the motor representation of the observer and the actor, emphasizing shared goals or action plans across the two actors (Wilson and Knoblich, 2005).

Although there is an extensive literature on the mirror system mechanisms involved in observation of actions (e.g., Fadiga et al., 1995; Decety et al., 1997; Johnson-Frey et al., 2003; Iacoboni et al., 2005), the problem posed by this review is somewhat different. In most cases of explicit or implicit use of other's affordances and of spontaneous use of another's viewpoint in perspective taking, there is no overt movement of the other agent. It is possible that observers use intrapersonal motor resonance to not only emulate actions, but also to infer and predict future actions (Wilson and Knoblich, 2005; Sebanz et al., 2006). Specifically, experience and capabilities or current bodily state could be used to predict the actions of others. Bosbach et al. (2005) showed the importance of one's proprioceptive body information on action understanding by demonstrating that individuals with impaired sense of touch and proprioception failed to understand another's expectation of weight when observing the action (see also Reed and Farah, 1995; Daems and Verfaillie, 1999 for posture-based effects). Knoblich and colleague's proposal that the observer serves as an initial model for understanding and predicting action could explain some of the results discussed so far. For example, the influence of wearing ankle weights on judging other's jumping ability would relate one's own action capability to judgments for another's capabilities (Ramenzoni et al., 2008a). Likewise, the capability of another agent to reach or not reach a mug could influence one's own likelihood of reaching the mug, leading to more or less priming of the motor system (Costantini et al., 2011). This claim is supported by more recent work (Cardellicchio et al., 2013) which used transcranial magnetic stimulation (TMS) to record the motor-evoked potentials (MEPs) of observers. In a virtual environment display, a mug was presented either within or outside of the observer's reaching space and within or outside of an agent's reaching space. Highest MEPs were measured when the mug was within either the observer's reaching space or the agent's reaching space, compared to when the mug was outside of the observer's reaching space or close to a non-body cylinder (which took the place of the avatar/agent). Finally, in joint actions, there could be neural representations for action based on each actor's capabilities that mutually activate in order to support complementary actions.

### Information-based accounts

An alternative account of self-other interactions comes from the ecological viewpoint, emphasizing the direct information about the environment available to the viewer. As mentioned in the introduction, this account is not necessarily exclusive of the motor resonance account, but it emphasizes different aspects of the processes of social perception-action. As described earlier, Ramenzoni et al. (2008b) found that viewers used eye-height scaled information to judge accurately what others could reach, suggesting that judging other's affordances relies on viewer-scaled optical information. Indeed, Ramenzoni et al. (2010) argued that the motor resonance account proposes a “strong dependency on the observer's own action capabilities” (p. 1117) that is not necessarily supported by the empirical findings. Accounts based in motor simulation place an emphasis on the attributes of the perceiver in judging other's affordances, rather than the situated perceptual information available to the other agent. In many cases, studies of judging other's affordances have shown the importance of the perceptual information available to the agent, in contrast to a reliance on the perceiver's capabilities.

A possible mechanism for this direct use of environmental information may be explained by the *synergistic* approach (Riley et al., 2011). In this approach, observers are thought to be able to coordinate actions with others through a process of reducing each other's degrees of freedom in movement (dimensional compression) and reacting to the movements of one another (reciprocal compensation) to create a single coordinated system (Riley et al., 2011). The synergistic approach extends the work of Nikolai Bernstein in motor coordination. Bernstein identified that one major problem for any movement system, such as the human body, is in regulating all the possible degrees of freedom inherent to it (e.g., joints, muscle extension/flexion, etc.). Bernstein (1967) proposed that these degrees of freedom may couple together to create a synergy. By allowing for synergies, the overall degrees of freedom are reduced allowing the movement system to work as a single unified system. In applying the synergistic approach at the interpersonal level, Riley et al. (2011), consider how two individuals couple their actions to produce a synergy that ultimately constrains the degrees of freedom in the movement of each individual. Because viewers have access to concurrent visual information from multiple viewpoints and can judge affordances for another with respect to the other's bodily information in the context of the environment, they can also interpersonally coordinate actions. Overall, the synergistic approach describes a process that allows observers to couple their movements with those of others, which gives rise to dynamic changes that are not independent in the two systems (see Kugler and Turvey, 1987; Turvey and Carello, 1996).

The synergistic approach may better explain phenomena such as understanding the interpersonal exchange in conversations (Condon and Ogston, 1971) and similar affect in interactions between mothers and their children (Cohn and Tronick, 1988) than the motor resonance approach. More related to the current paper, Ramenzoni (2008) asked participants to coordinate holding a stick inside a hollowed circle. When circle size was varied, the task became more or less difficult and as a result, participants' hand and torso movements were more or less coordinated (see also Riley et al., 2011). The main difference between this approach and that of Sebanz et al. (2006) is the claim that actors' movements in a coordinated action are not independent of one another, rather they coordinate to form a new entity with which to judge affordances. As such, the motor resonance approach may predict dimensional compression, but it cannot account for reciprocal compensation due to the assumption that the mirror neurons systems of two individuals are independent of one another (Riley et al., 2011). In addition, this approach does not focus on fixed neurological structures causing the activity of other structures; rather it focuses on the functionality that arises when many neurological structures interact or couple together, reflecting Bernstein's (1967) original approach to understanding motor coordination.

## Conclusions

Perceiving other's affordances and spatial perspective taking are two abilities that have traditionally been studied in the domains of perception and spatial cognition, respectively. While typically considered separate abilities, they share a common conceptual foundation of relating self and other perspectives in some way. An observer must determine how another agent can act or see the world. While these are skills that are important fundamentally for an understanding of our spatial environment, we argue that when considered together, they provide a basis for a broader social function of human behavior prediction critical to our social coordination with others. In this paper we aimed to provide a review of the work carried out on other's affordances and perspective taking to show how they are related in the service of understanding both the actions and intentions of others.

Judging other's affordances is a means to determine capability for future action. The literature reviewed shows that in circumstances of a single other agent, or in dyads, observers are relatively good at perceiving affordances for others when provided with enough information to scale judgments to the other's body. However, we have proposed that these laboratory-based affordance judgments are typically more specified in terms of an action-goal than what occurs in the real world where the other's goal may not be as specified. To solve this problem and identify another's intentions, the ability of spatial perspective taking may come into play, allowing an observer to further define the intention and goal of the other actor. Support for these two components as complementary processes comes from an analysis of the similarities between the two, on both computational and neural mechanism levels.

An analysis of frames of reference recruited shows us that there are at least three possible frameworks used. The viewer may use their own egocentric frame (as used in judgments of self-affordances), which may also include a reliance on their own possibilities for action when judging for others; alternatively, a viewer's egocentric frame of reference may be transformed onto the other's frame of reference, aligning the self and other reference frames, typically used in PT-2 tasks; finally, the viewer may simply use an allocentric frame of reference, computing the relationship between the other and the target object/environment. Current work suggests more overlap in the allocentric computation used in perceiving other's affordances and PT-1; however, more work is needed to determine whether egocentric spatial transformations may be involved in some affordance judgments. Future studies addressing this question could assess the possible transformation of the egocentric frame by measuring angular disparity effects during explicit or implicit affordance judgments with respect to other agents.

An analysis of motor resonance theory suggests that the sensorimotor mechanisms supporting some forms of perspective taking and perceiving other's affordances may overlap. This is particularly apparent in circumstances in which there is no available visual information to make judgments of affordances or perspective—e.g., insufficient information about kinematics or the need for updating of spatial relations in a viewer-centered framework. In these cases, viewers may use motor simulation to judge the capabilities or perspective of others. Furthermore, the spontaneous and mutual influence of another agent and the self, seen in both affordance judgments and perspective taking, also is consistent with shared spatial and proprioceptive information among two people, as well as shared motor processing. In all, we suggest that judging affordances and spatial perspective rely on a combination of direct visual information and motor resonance.

Finally, we have considered how the broader goal of social cognition could be served by two spatial processes, but it is also important to consider the possibility of the inverse. Does social context itself moderate the abilities of perceiving other's affordances and perspective? The underlying rationale is that in order to perform a spatial switch of perspectives, one must understand that other agents have different perspectives. Thus, having a “theory of mind” could be a prerequisite to spatial perspective taking. The influence of social skills on spatial perspective taking has been shown in a number of ways. First, individuals with autism spectrum disorder (ASD) have been studied as a population that is defined with social impairment. Hamilton et al. (2009) showed a subtle distinction between performance on two mental rotation tasks in ASD children, finding impairment on a perspective rotation condition in which the decision required was with respect to what another person could see, but not on an object-rotation condition. Shelton et al. (2012) investigated the influence of social skills on perspective taking by testing a healthy non-clinical population, but using a questionnaire to assess traits of ASD. In a version of Piaget's three mountain task, they asked observers to choose a picture of a display as it would appear from another perspective. The location of the other's perspective was indicated either by a triangle, camera, or a doll. They found that perspective taking performance was modulated by social skills, but only for the doll, such that better social skills were associated with better perspective taking. Similarly, Kessler and Wang (2012) found that differences in perspective taking emerged as a function of both sex and social skills.

While not directly the same task as the mostly static affordance or spatial judgments focused on in this paper, there is also a recent literature on the influence of social context of others on executed actions. For example, reach-to-grasp kinematics are different when passing an object to a partner compared to placing it in a new location (Becchio et al., 2008) and implicit social requests for an object have been shown to override an initial motor plan (Sartori et al., 2009). Together, this work emphasizes the importance of social context on action planning and the flexibility in online adjustments in action that occur with potential social interactions.

Clearly, there is a need to consider what may seem to be disparate areas of research to understand complex human behaviors, such as social coordination and joint action. This review provides one example for which research on two distinct spatial processes—judgments of others' affordances and spatial perspective taking—may be examined to elucidate potential mechanisms for more complex behaviors.

### Conflict of interest statement

The authors declare that the research was conducted in the absence of any commercial or financial relationships that could be construed as a potential conflict of interest.
